# Biodegradable magnesium‐based biomaterials: An overview of challenges and opportunities

**DOI:** 10.1002/mco2.59

**Published:** 2021-04-08

**Authors:** Shukufe Amukarimi, Masoud Mozafari

**Affiliations:** ^1^ Department of Tissue Engineering & Regenerative Medicine, Faculty of Advanced Technologies in Medicine Iran University of Medical Sciences (IUMS) Tehran Iran

**Keywords:** biodegradability, biomaterials, magnesium, medical device, tissue engineering

## Abstract

As promising biodegradable materials with nontoxic degradation products, magnesium (Mg) and its alloys have received more and more attention in the biomedical field very recently. Having excellent biocompatibility and unique mechanical properties, magnesium‐based alloys currently cover a broad range of applications in the biomedical field. The use of Mg‐based biomedical devices eliminates the need for biomaterial removal surgery after the healing process and reduces adverse effects induced by the implantation of permanent biomaterials. However, the high corrosion rate of Mg‐based implants leads to unexpected degradation, structural failure, hydrogen evolution, alkalization, and cytotoxicity. To overcome these limitations, alloying Mg with suitable alloying elements and surface treatment come highly recommended. In this area, open questions remain on the behavior of Mg‐based biomaterials in the human body and the effects of different factors that have resulted in these challenges. In addition to that, many techniques are yet to be verified to turn these challenges into opportunities. Accordingly, this article aims to review major challenges and opportunities for Mg‐based biomaterials to minimize the challenges for the development of novel biomaterials made of Mg and its alloys.

## Graphical abstract

Magnesium (Mg)‐based alloys have shifted into the focus as a new generation of degradable biomaterials for medical applications. Because biodegradable Mg‐based alloys possess excellent biocompatibility and unique mechanical properties, deploying these biomaterials eliminates the need for biomaterial removal surgery after the healing process and diminishes adverse effects induced by implantation of permanent implants. This article reviews the main challenges following the implantation of Mg‐based biomaterials in the human body. It also introduces a broad overview of opportunities to minimize the challenges for the development of advanced biomaterials made from Mg and its alloys.

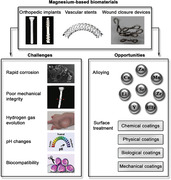

## 1 INTRODUCTION

To date, a significant number of metallic biomaterials have been manufactured for load‐bearing applications, mainly for the replacement of injured hard tissues, in the biomedical field.^1^ In spite of the fact that the traditional paradigm of metallic implants needed metals with high corrosion resistance in the human body,^2^ a fundamental disadvantage of them for medical applications was the lack of biodegradability, causing concern about lifelong toxicity.^3^, ^4^ Lately, progress in the field of biomedical engineering indicates a positive role of biodegradable metals believed to gradually corrode and degrade in physiological environments with a proper host response obtained from liberated degradation products.^5^, ^6^, ^7^ In reality, biodegradable metals are designed to fulfill the mission of supporting tissues and accelerating the healing process.^8^, ^9^ Therefore, no second surgical procedure is required for implant removal, decreasing extra costs, the morbidity of the patient, and the risk of new symptoms.^10^, ^11^, ^12^ Till now, biodegradable metals for biomedical applications can be classified into three subgroups: iron (Fe), zinc (Zn), and magnesium (Mg), along with their alloys.^5^, ^13^

Among biodegradable metals, Fe, with the highest mechanical strength and elastic modulus, has mechanical properties similar to traditional permanent metallic biomaterials made of stainless steel, not natural bones. Therefore, having an explicit contrast between elastic moduli of orthopedic biomaterial and injured bone, the implantation of Fe‐based orthopedic implants might result in elastic mismatches and trigger stress shielding.^5^, ^14^ Although unlike the permanent implants, Fe degrades over time, its degradation process is considerably slow in comparison with other biodegradable metals, thereby acting just like that of permanent implants. For this reason, to accelerate the degradation rate of this metal, a slight amount of manganese (Mn) is usually added into pure Fe to create micro‐galvanic corrosion sites and accelerate its degradation rate.^13^, ^15^ Another biodegradable metal gaining research and clinical interest in the recent past is zinc. Zn with a standard electrode potential (–0.763 V) between that of Fe (–0.44 V) and Mg (–2.363 V)^16^ has near‐ideal corrosion resistance owing to passive layers of moderate stability generated by degradation products.^17^, ^18^, ^19^ However, implants made of pure Zn may not be suitable for most medical applications as they suffer from poor plasticity (*ɛ* < 0.25%) and low strength (*σ*_UTS_ ∼30 Mpa).^13^, ^17^, ^20^ Table 1 shows a comparison of the advantages and disadvantages of common metallic biomaterials used in the human body.

**TABLE 1:** Main characteristics of metallic biomaterials used in the human body

Materials	Advantages	Disadvantages	References
Stainless steel	High wear resistance, low cost, easily available, acceptable biocompatibility	Allergic reaction,^a^ much higher modulus than bone, lack of biodegradability	^34^, ^35^, ^36^
Co‐based alloys	High wear and corrosion resistance, fatigue strength	Biologically toxic,^a^ much higher modulus than bone, expensive, lack of biodegradability	^36^, ^37^
Ti‐based alloys	High biocompatibility and corrosion resistance, fatigue strength, light weight, low Young's modulus	Poor tribological properties, lack of biodegradability	^38^, ^39^, ^40^
Fe‐based alloys	Trace element, biodegradable, high radial strength,^b^ superior mechanical properties	Very slow corrosion process,^c^ excessively slow degradation rate, inadequate match of mechanical properties to those of a natural bone, magnetic nature^d^	^4^, ^41^, ^42^
Zn‐based alloys	Trace element, biodegradable, biocompatible, easy to cast and process, good machinability, moderate corrosion resistance	Low strength and plasticity	^17^, ^43^
Mg‐based alloys	Trace element, biodegradable, biocompatible, having mechanical properties similar to those of bone	Low corrosion resistance against living body circumstances, rapid loss of mechanical integrity, hydrogen evolution and alkalization during degradation	^29^, ^30^

^a^ Ni, Cr, and Co can induce allergic reactions in humans.

^b^ If cardiovascular stents show high radial strength, they will permit the production of more ductile structures and very thin stent struts. Therefore, it will be easier to deploy into the artery.

^c^ Although having high corrosion resistance is a positive attribute, having very high corrosion resistance might lead to slow degradation of the implant and the phenomena of stress shielding. It may also prevent tissue regeneration.

^d^ In general, the presence of Fe in surgical biomaterials might hinder magnetic resonance imaging used widely to visualize the physiological procedures and the anatomy of the patients during diagnosis and healing process. Moreover, exposure to strong magnetic fields might increase the temperature of the biomaterial, changing the position or shape of the biomaterial.

According to Table 1, Mg is much more suited for use as a biodegradable biomaterial not only for its similar mechanical properties to those of bone but also for its biocompatibility.^21^, ^22^, ^23^ In fact, Mg is a necessary nutrient that the body requires to stay healthy: it fulfills various intracellular physiological functions, stimulates bone growth, improves cell adhesion to biomaterials, assists the differentiation and biomineralization of osteoblasts, and lowers the risk of osteoporosis and coronary artery disease.^24^, ^25^, ^26^, ^27^ Moreover, the nontoxic magnesium degradation products are not generally a cause for concern, disorder, inflammation, and allergic reactions in vivo, and Mg^2+^ amount in excess can be easily excreted into the urine, thus maintaining the same amount of Mg^2+^ in the body.^28^ However, the development and industrialization of Mg‐based biomaterials are just at the beginning. This is because the high corrosion rate of Mg and its alloys in the physiological environment can result in unexpected degradation, loss of mechanical integrity at the initial stages of degradation, and implant failure before the healing process.^12^, ^29^, ^30^ Furthermore, the production of some corrosion products such as hydrogen gases and hydroxide ions can influence biocompatibility. As for illustration, hydrogen evolved for corroded Mg can accumulate in the form of gas cavities in the surrounding tissue, resulting in the separation of tissue layers. Hydroxide ions can cause surface alkalization and might harm cells.^31^, ^32^ Therefore, a great deal of research is required to control the degradation behavior of Mg and its alloys in vivo and add more advanced properties and functions to Mg‐based biomaterials. In this field, alloying Mg with nontoxic alloying elements and coating the surface of Mg‐based implants are reported to be beneficial.^12^, ^30^, ^33^ The present review article focuses on the challenges and opportunities of magnesium alloys after introducing Mg‐based biomaterials.

## 2 Mg AND ITS ALLOYS AS BIODEGRADABLE BIOMATERIALS

Biodegradable magnesium alloys are candidate materials for load‐bearing applications. Being biodegradable, these materials save a second surgery for the implant removal and minimizes the influences of the left implant in the body for some time when they are no longer required for medical purposes.^44^, ^45^, ^46^ Medical application of magnesium was first announced in 1878 after Edward C. Hause utilized Mg wires as ligatures for bleeding vessels, and since then, extensive studies have revealed the positive role of Mg alloys in medicine and surgery for numerous applications.^47^

**FIGURE 1:**
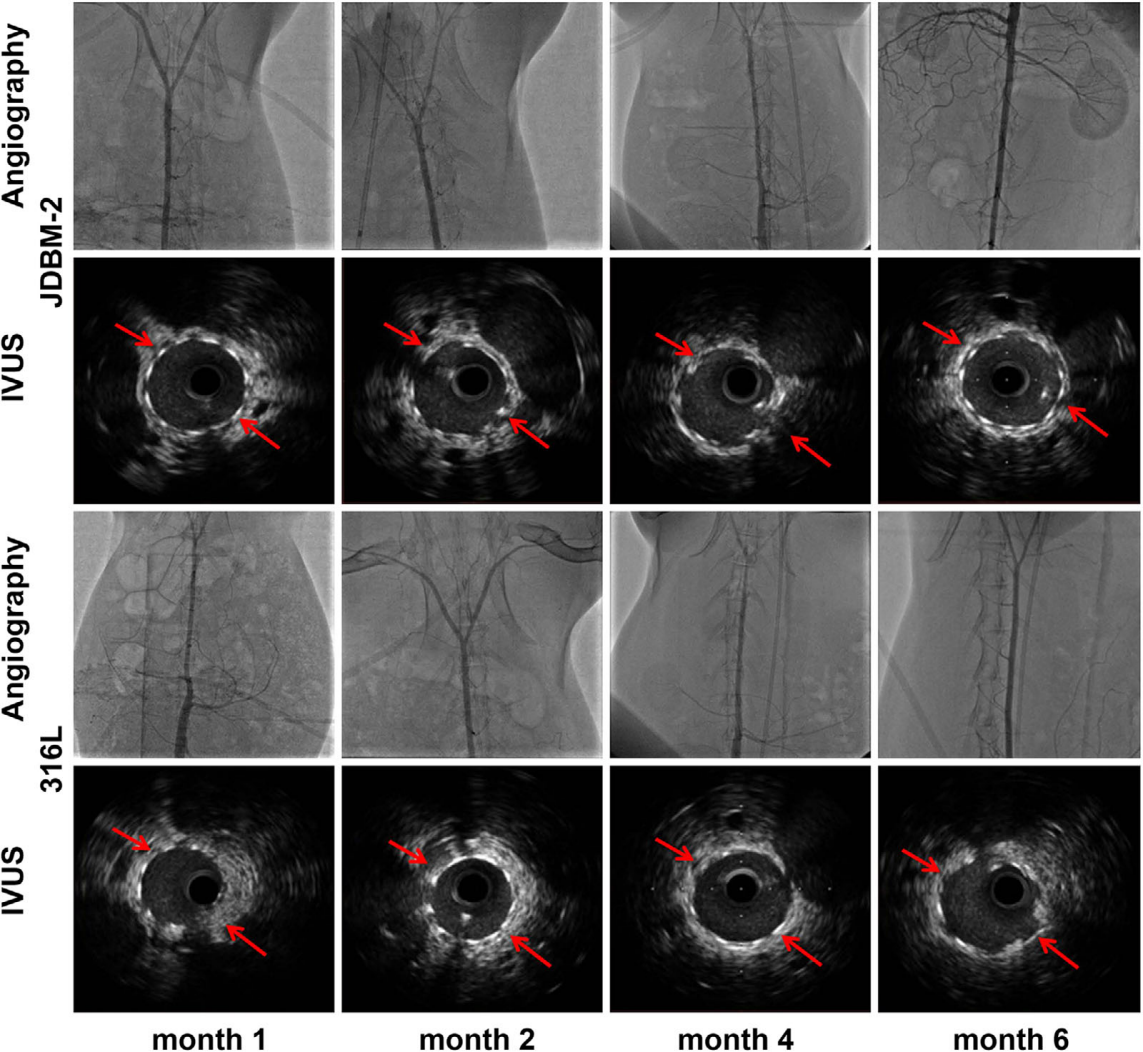
In vivo angiography and the follow‐up IVUS results. The in vivo aortic angiography indicating no in‐stent restenosis and late thrombogenesis in the JDBM‐2 alloy and 316L stainless steel (SS) (served as negative control) vascular stents after implanting in rabbit abdominal aorta for 1, 2, 4, and 6 months. The corresponding follow‐up IVUS photographs demonstrating the longitudinal reconstruction of the abdominal aorta after the JDBM‐2 alloy and 316L SS stenting. Enhanced vessel size and lumen patency at various implantation time with the absence of neointimal hyperplasia show that JDBM‐2 alloy can be a promising alloy for vascular stent application. Adopted with permission^54^

The first group of Mg biomaterials is Mg‐based vascular stents. Mg is a potential candidate for use as a vascular stent^48^, ^49^ because it helps the regulation of heart rhythm, enhances blood flow, hinders platelet activation, prevents blood vessels from constricting, lowers blood pressure, and relaxes vascular smooth muscle cells.^50^, ^51^ It is also important to note that Mg supply may result in a risk reduction toward coronary artery disease.^52^, ^53^ Mao et al.^54^ implanted Mg–2.2Nd–0.1Zn–0.4Zr (denoted as JDBM‐2) vascular stent in rabbit abdominal aorta. The in vivo results indicated up to 6‐month mechanical integrity and good biocompatibility of the JDBM‐2 stent. Figure 1 shows that the JDBM‐2 stent was entirely expanded and well apposed to the vessel wall with no sign of elastic recoil, fracture, late thrombogenesis, and in‐stent restenosis. Accordingly, the JDBM‐2 Mg alloy can be considered as a suitable candidate for vascular stent application. However, inadequate corrosion resistance of several Mg‐based alloys is still challenging for this application because short‐term support of fewer than 6 months can result in premature loss of mechanical integrity and stent failure.^55^

**FIGURE 2:**
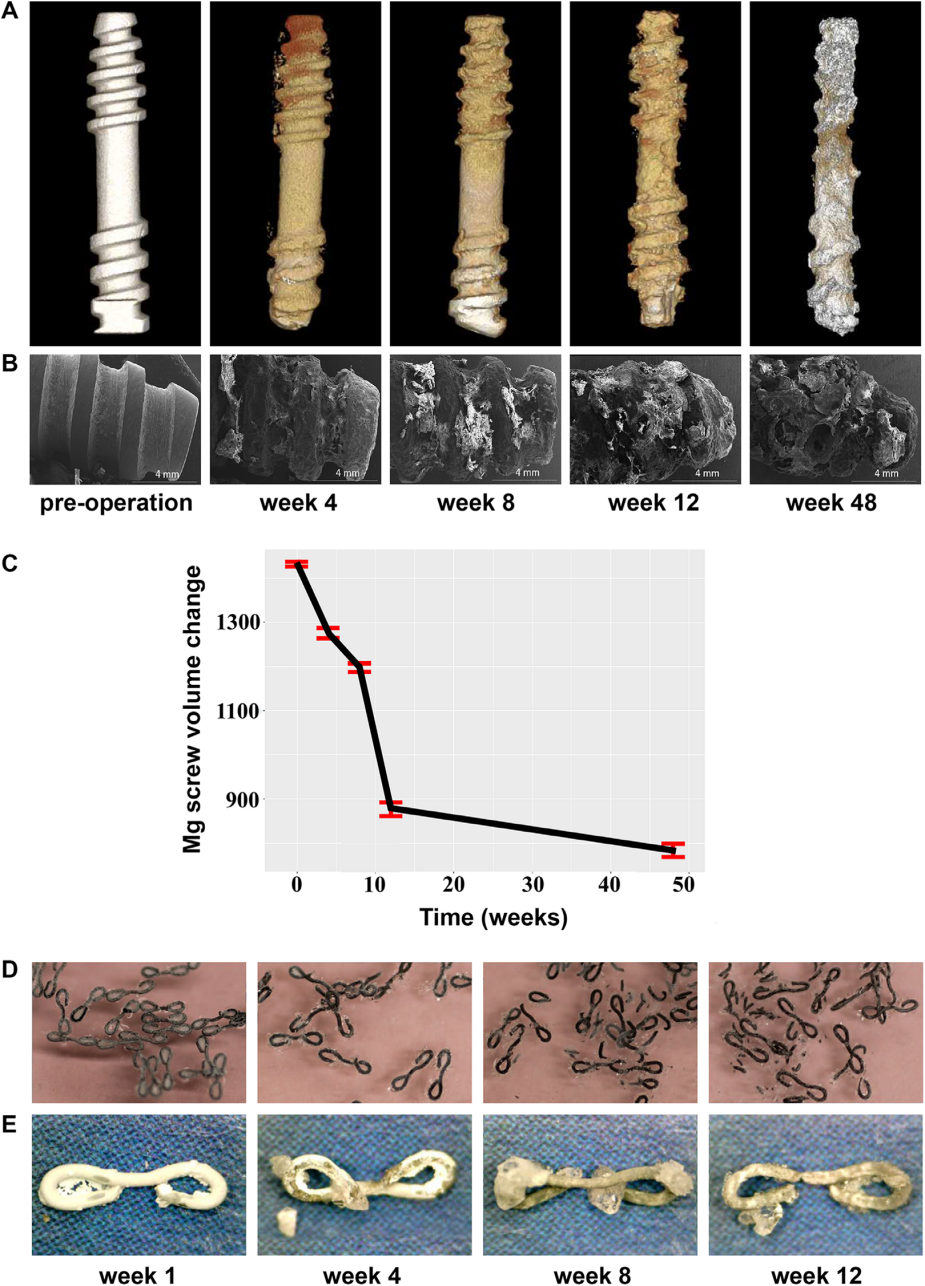
The degradation process of Mg biomaterials. (A) Micro‐CT photographs of the implanted high‐purity magnesium screw in goats. (B) The SEM morphologies of the screws after 4, 8, 12, and 48 weeks implantation. (C) Quantification of the degradation of high‐purity Mg screws as evaluated by the reduction in surface area of the screws at the given time points, showing that the main body of the screws and the threads were significantly degraded at a period of 48‐week after surgery. At this time of implantation, the femoral head of the goat with the expected shape was healed. Overall, high‐purity Mg screws indicated adequate mechanical integrity and satisfactory degradation kinetics compatible with the healing procedure. (D) Macroscopic display of the FAsorbMg staples while immersing in the artificial intestinal juice and (E) when staples were removed from the artificial intestinal juice at 1, 4, 8, and 12 weeks. A white layer of corrosion products formed on the surfaces of the staples. H_2_ gases produced from the fast corrosion of Mg were formed in the first week of immersion assay only in trace amounts, and most staples kept their shapes and mechanical integrity until at least the fourth week. In vitro results showed satisfactory biodegradation behavior, mechanical durability, and biocompatibility of Mg alloy staples. (A‐C) Adopted with permission.^72^ (D and E) Adopted with permission^80^

Mg biomaterials of the second group are Mg‐based orthopedic implants used widely for the replacement and regeneration of damaged hard tissues.^56^, ^57^, ^58^ Orthopedic implants are desired to have mechanical properties similar to those of natural bone to avoid stress shielding effect. Stress shielding refers to the reduction of bone strength and density because of bearing a higher proportion of the applied load and stress by an orthopedic implant from the adjacent bone. Among different metallic biomaterials, Mg is known to have density and elastic modulus most similar to cortical bone (Mg: 1.78 g/cm,^3^ 40–45 GPa; Cortical bone: 1.8 g/cm,^3^ 10–27 GPa).^59^ Studies have shown that using Mg as an orthopedic biomaterial can promote bone reconstruction and accelerate the healing process.^60^ As a result, Mg alloys are suitable materials for orthopedic applications.^61^, ^62^, ^63^ Orthopedic biomaterials include bone and cartilage scaffolds,^64^, ^65^ as well as various fixation devices such as plate–screw fixation systems,^66^, ^67^ nails,^68^, ^69^ pins,^70^ and wires.^71^ Huang et al.^72^ implanted high‐purity Mg screws into goats for the fixation of femoral neck fractures. The results indicated that the bone tissues of the femoral head and neck were successfully healed by the implantation of pure Mg screws degraded compatible with bone reconstruction. Figure 2A–C presents the results of the degradation process of pure Mg screws. According to these figures, there were no major changes in the appearance of screws 4‐week postimplantation; however, after 48 weeks, the screw threads, along with the main body, considerably degraded. At this time, the femoral head of the goat was reconstructed in the expected shape.

The last group of Mg‐based biomaterials is wound closure devices such as Mg ligature,^73^ clips,^74^ staples,^75^ Velox CDs,^11^ and newly surgical tacks.^76^ These biomaterials are designed to aid in closing wounds by manipulating wound edges closer together for a certain period, then they are entirely absorbed by the patient's body during a couple of weeks without affecting the characteristics of the connected tissues.^4^, ^77^, ^78^ Surgical Mg‐based wound closure biomaterials not only can provide the best healing results but also can be easily exerted onto wounds. This is because they have the ability to tolerate high vibration frequencies and shearing stresses during phonation.^79^ Amano et al.^80^ evaluated the safety and feasibility of biodegradable Mg alloy (Mg 2 wt%, Nd 1 wt%, Y, FAsorbMg™) staples using finite element analysis (FEA) and in vitro and in vivo experiments. FEA demonstrated the appropriate stress distribution of the FAsorbMg staples while stapling and maintaining closure. The in vitro experiment using artificial intestinal juice indicated sufficient biocompatibility, degradation behavior, and mechanical durability of staples because most of them kept their mechanical integrity until at least the fourth week (Figures 2D and 2E). The in vivo test was carried out in porcine intestinal anastomoses, showing neither technical failure nor such complications as anastomotic leakage, adhesion, or hematoma.

## 3 CHALLENGES OF Mg‐BASED BIOMATERIALS

### 3.1 Rapid corrosion of Mg‐based biomaterials in the physiological environments

Despite the fact that the ability of Mg and its alloys to degrade has led to plenty of medical applications over the past few years, the corrosion behavior of Mg‐based implants is of primary concern because the lowest standard electrode potential of Mg (‒2.363 V) contributes to a high corrosion rate.^81^, ^82^ However, Mg implants are able to cover themselves with a layer of degradation products, which creates a kinetic impediment on the surface of the implants, and physically avoids migration of Mg^2+^ cations from the surface of the Mg implant to the aqueous environment.^83^ Consequently, Mg and its alloys oxidize in the presence of water owing to the low thermodynamic stability. In fact, when Mg is placed in water, the anodic reaction of Mg occurs, leading to the production of Mg^2+^ cations from the surface of Mg, as is presented in Equation (1). At the same time, the cathodic reaction occurs while protons reduce at the cathode, thereby liberating H_2_ gas (formation of gas cavities) and OH^‒^ ions (surface alkalization), as is expressed in Equation (2). Finally, at the corrosion potential, a thin film of Mg(OH)_2_ covers the Mg surface (Equation 3). The precipitation of Mg(OH)_2_ attaches to the surface and provides a passive layer. However, the formed Mg(OH)_2_ layer is quite loose, so it cannot protect magnesium against corrosion completely. Moreover, the production of H_2_ gas during degradation at the corrosion sites can separate the deposited Mg(OH)_2_ pieces from the surface of the Mg substrate and avoid covering a uniform and stable Mg(OH)_2_ film, which means that Mg degradation is not self‐inhibited. Therefore, it continues until the complete degradation of the Mg biomaterial.^84^, ^85^, ^86^

(1) $$\mathrm{Anodic}\;\mathrm{reaction}:\;\mathrm{Mg}\to Mg^{2+}+2e^{-}$$

(2) $$\mathrm{Cathodic}\;\mathrm{reaction}:2H_{2}O+2e^{-}\to 2OH^{-}+H_{2}$$

(3) $$\begin{array}{ccc} \mathrm{Product}\;\mathrm{formation}:\mathrm{Mg}\left(s\right)+2H_{2}O\left(l\right) & \to & \mathrm{Mg}{\left(\mathrm{OH}\right)}_{2}\left(s\right)\\ & & +\;H_{2}\left(g\right) \end{array}$$

On the other side, the corrosion performance of Mg biomaterials in the human body is more complicated. As is well‐known, blood plasma contains water, proteins, ions, and so forth. The principal plasma ions are Na^+^, K^+^, Ca^2+^, Cl^‒^, HCO_3_^–^, HPO_4_^2‒^, PO_4_^3‒^, SO_4_^2‒^, and Mg^2+^.^87^, ^88^ Chloride ions are aggressive ions that disrupt the protective layers on the surface of Mg. The corrosion resistance of Mg and its alloys, therefore, decreases with increasing chloride concentration.^89^ Because PO_4_^3‒^ ions are well‐known corrosion impeders, the presence of these ions can lead to an increase in the corrosion resistance of Mg implants.^90^ Generally, phosphates and carbonates might increase the production of protective corrosion product films on the surface of Mg, resulting in better corrosion resistance.^91^ As well as ions, organic components such as cells, biomolecules, bacteria, and proteins can affect the mechanism and kinetics of corrosion of Mg biomaterials. The adhesion of cells and the growth of endothelial cells and osteoblasts on the Mg surface can decelerate the corrosion rate in vivo. Proteins can be absorbed on the surface of Mg implants leading to cellular attachment, migration, proliferation, and the formation of complex metal ions. Moreover, they can act as an inhibitor, promoter, or both depending on the time of Mg implant corrosion.^92^

As the corrosion rate increases exponentially with higher temperatures, the temperature of the human body (37°C) can increase the rate of corrosion of magnesium compared to room temperature. Moreover, the temperature can influence the precipitation of different degradation products in a physiological solution. For example, the solubility of some precipitates on the surface of a corroding Mg implant is temperature dependent.^93^ Apart from temperature, the pH value of an electrolyte that Mg is dissolving can change the corrosion resistance of magnesium‐based implants. As Mr. Pourbaix reported, biodegradable magnesium is significantly susceptible to corrosion in most inorganic acidic, neutral, and slightly alkaline solutions with a speed that reduces as the pH increases.^94^ From another point of view, the production of OH^–^ while the corrosion of Mg can shift the local pH into the alkaline region.^90^

The hydrodynamic condition of the human body influences local surface chemistry, corrosion, and biological behavior. For instance, blood flow surrounding Mg biomaterials can avoid the growth of corrosion products by transporting them away from the implant surface, thereby increasing the corrosion rate of the Mg biomaterial.^93^ By way of illustration, as Mg vascular stents are exposed to the blood flow in the arteries, they might be more susceptible to rapid corrosion compared to cartilage scaffolds made of Mg alloys because there are no blood vessels in cartilage.

**FIGURE 3:**
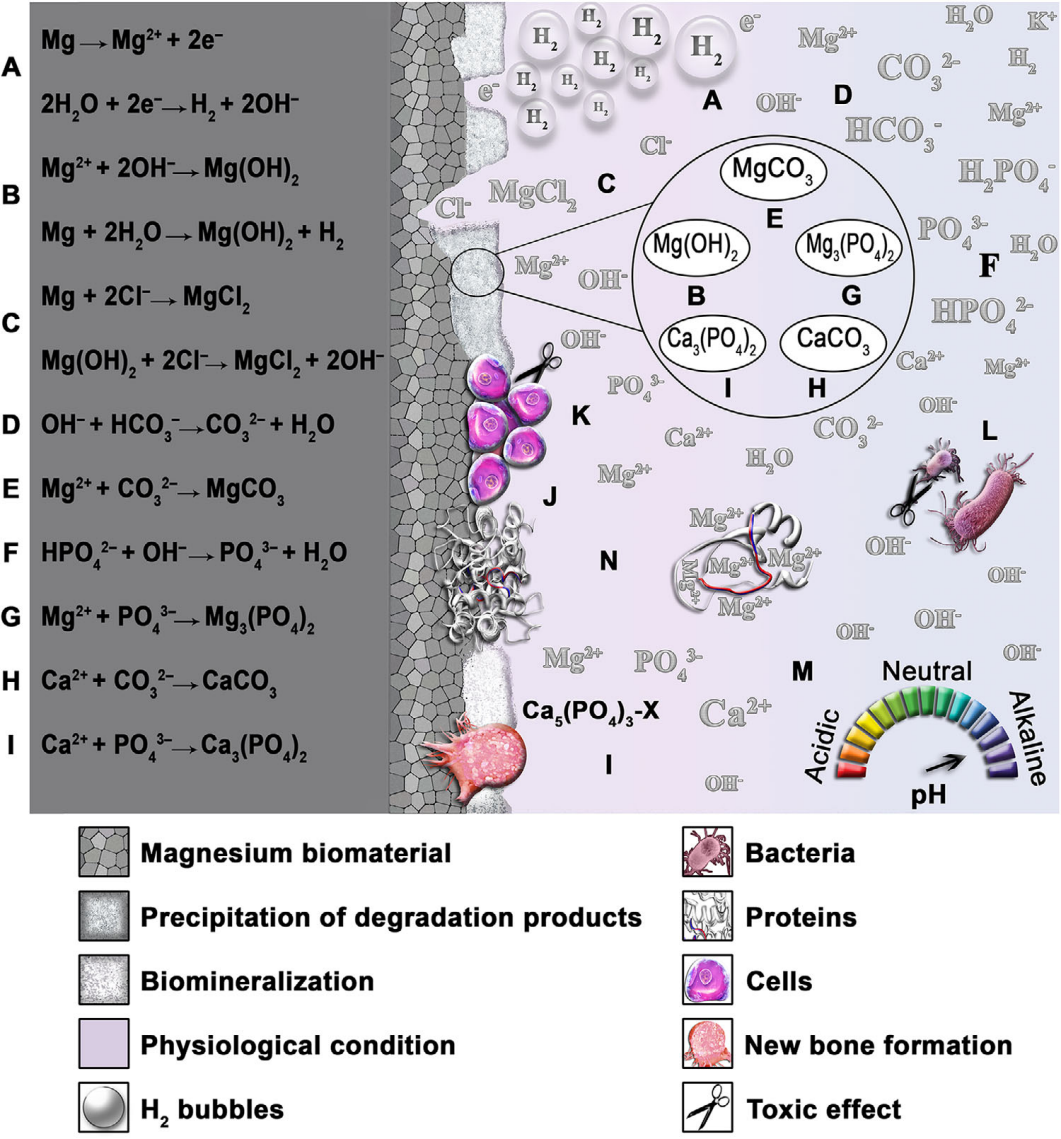
Schematic illustration of the corrosion behavior of biodegradable magnesium biomaterial in physiological conditions and several possible chemical reactions. (A) The corrosion of Mg results in the production of Mg^2+^ cations, H_2_ bubbles, and OH^‒^ ions. (B) A white compound with low solubility in water chemically named Mg(OH)_2_ forms on the surface of Mg. (C) Chloride ions attack the surface of Mg and disrupt the protective layers on that, resulting in the formation of MgCl_2_ and a higher corrosion rate. (D) OH^‒^ ions react with bicarbonate, thus producing carbonate ions and water. (E) MgCO_3_ formation owing to the presence of Mg^2+^ and carbonate ions. (F) HPO_4_^2‒^ reacts with OH^‒^ ions and generates phosphate ions and H_2_O. (G) Phosphate ions react with Mg^2+^ cations, thereby producing Mg_3_(PO_4_)_2_. (H) Calcium carbonate is formed due to the presence of Ca^2+^ and carbonate ions. (I) Calcium phosphate biomineralization, because of the presence of calcium and phosphate. Several possible corrosion products here are tricalcium phosphate and hydroxyapatite (HA), besides X can be, OH, Cl, and so forth. (J) Adherence of cells and proteins to the surface, thereby slowing down the corrosion rate. (K) The hydroxide ions might harm cells. (L) An increase of hydroxide ions leads to an antibacterial influence. (M) As the level of OH^–^ increases, the more basic, or alkaline, the physiological solution becomes, which decelerates the corrosion rate of Mg. (N) The adsorption of proteins on the surface of the Mg implant is followed by cellular attachment, migration, proliferation, and complex Mg^2+^ ions. (Source: Authors)

The abovementioned parameters, as well as many other unknown factors, can influence the corrosion behavior of Mg‐based biomaterials in numerous ways, resulting in a very complex corrosion scenario in the human body, which is still far from full understanding. Accordingly, in this field, an accurate prediction of the corrosion performance of Mg alloy implants within the human body needs exploring. Some possible interactions with a corroding Mg biomaterial in the body are depicted in Figure 3.

### 3.2 Loss of mechanical integrity of Mg‐based biomaterials due to high corrosion rate

In vivo experiments have demonstrated that the loss of mechanical integrity due to the high rate of degradation of Mg‐based biomaterials has limited their applications in the biomedical field. Actually, the uncontrollable degradation of Mg alloys leads to loss of initial strength, the transformation of pore size, changes in the weight of biomaterials, subsequently, and structural failure of the Mg biomaterial before fulfilling the required mechanical function.^95^ Because of that, the lifetime of Mg‐based biomaterials is considerably hard to predict. Because Mg alloys consist of various alloying elements and impurities, the corrosion behavior of Mg alloys is not often uniform. The reason for this is that nearly all of the alloying elements are nobler than Mg, and coupling Mg with nobler metals results in rapid and nonuniform corrosion performance of Mg implants. Therefore, the local loss of mechanical integrity of Mg alloy biomaterials occurs, making the prediction of durability substantially complicated.^93^ In general, the degradation of an ideal biodegradable biomaterial should proceed slowly to maintain mechanical integrity and give adequate time for the tissue to start healing. Then, the degradation rate should increase when the mechanical integrity declines.^96^ According to Li et al.,^97^ a coronary stent made of Mg should maintain its mechanical integrity to complete the vessel remodeling procedure for a period of 6–12 months. After that, it should degrade completely in 12–24 months. In addition to coronary stents, orthopedic biomaterials made of Mg should maintain their mechanical integrity to support new bone formation for a period of 3–4 months. However, investigations have revealed that the mechanical integrity of most of the currently researched Mg alloys drops quickly at the initial stage of degradation; therefore, they are not able to meet the clinical needs.^58^

**FIGURE 4:**
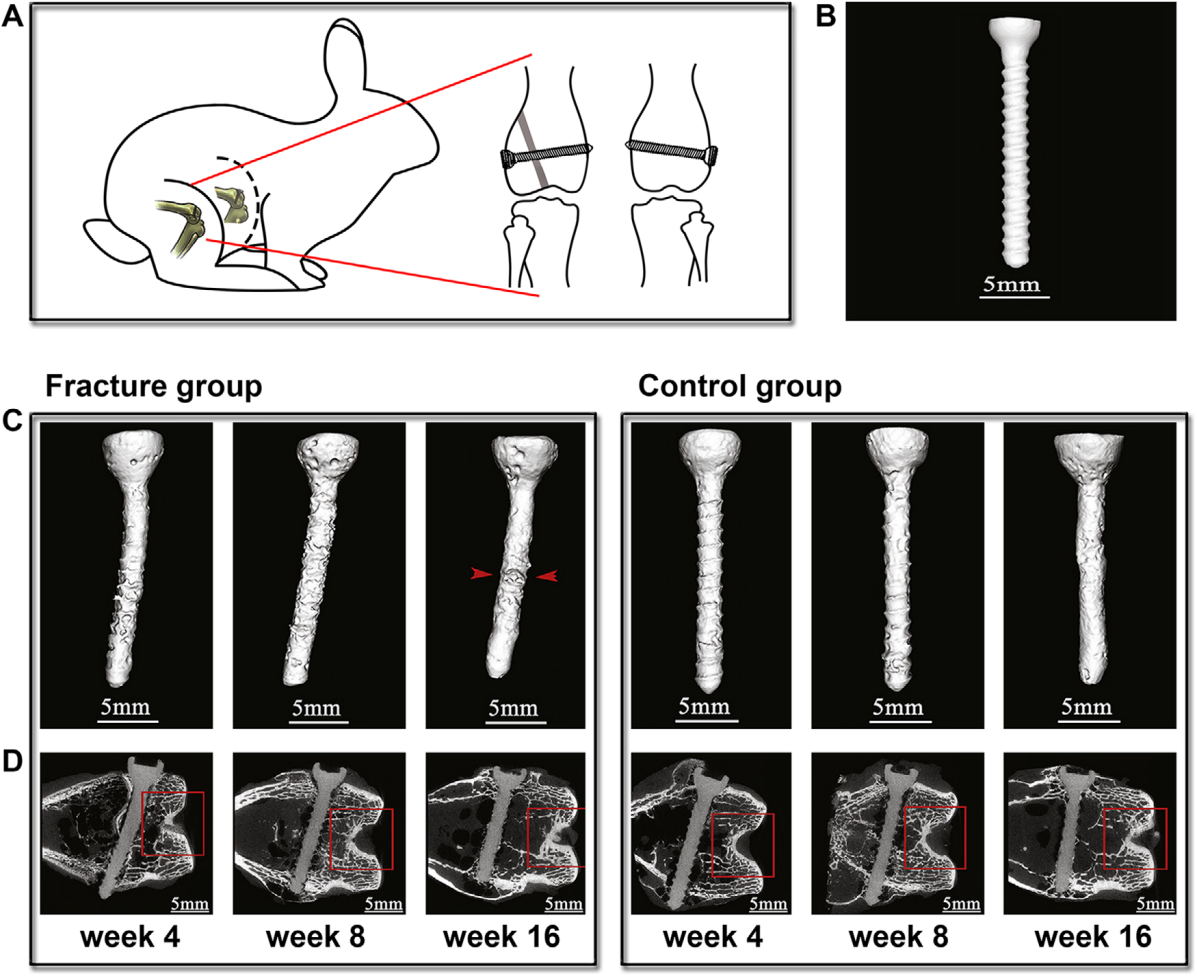
High‐purity magnesium screws for the fixation of femoral fracture and its implantation in the unscathed femoral. (A) Diagrammatic drawing of a rabbit displaying the high‐purity magnesium screws for fixing the femoral fracture (right leg of the rabbit) and the implantation of the same screw as a control sample in the (left leg of the rabbit). In the right leg, the gray part shows the fracture space of three millimeters. (B) A high‐purity Mg screw before surgery. (C) In vivo three‐dimensional photographs and (D), μ‐CT scans of high‐purity Mg screws both in the fracture and the control groups at 4, 8, and 16 weeks postimplantation. In the fracture group at 4 weeks, pure Mg screws had a bending angle at the part subjected to the fracture because of the mechanical stresses involved in fracture fixation. At 8 weeks postoperation, new tissue was formed in the gap. At 16 weeks, the formed bony bridge was developed, pure Mg screws were severely corroded, and mechanical integrity was reduced. Red arrowheads display the fracture gap. Adopted with permission^101^

Early failure of Mg‐based biomedical fixation devices might be as a result of stress corrosion cracking induced from the combined effect of corrosive physiological environment and mechanical loading.^98^, ^99^ Because Mg‐based orthopedic biomaterials experience loading owing to normal body movements such as running and walking and Mg‐based cardiovascular stents are subjected to cyclic loading due to heartbeat, the simultaneous action of cyclic loading and the corrosive physiological condition might result in corrosion‐assisted cracking.^100^ Han et al.^101^ implanted high‐purity magnesium screws in rabbits to repair a femoral fracture. The results indicated that 4 weeks postoperation, high‐purity Mg screws experienced bolt bending at the part exposed to fracture gap due to mechanical stress involved in fracture fixation, and 16 weeks after surgery, severe local corrosion was observed at the same part (Figure 4). This severe local corrosion is alleged to have been in the fracture group due to newly formed tissues in the gap because blood‐rich bone tissues stimulated the movement of ions on the surface of pure Mg screws. Although in the control group high‐purity Mg screws indicated corrosion in screw thread, no bending angle was seen in the control group.

It is remarkable to mention that every manufacturing process that decelerates the rate of degradation of Mg alloys can significantly enhance the mechanical integrity of Mg‐based biomaterials. For instance, Hou et al.^102^ evaluated the mechanical integrity of the rolled and the annealed ZX11 Mg alloys via tensile test after immersion assay. The results indicated that the deterioration in the mechanical integrity of the rolled ZX11 alloy was faster in comparison to the annealed one, which is in agreement with the higher degradation rate of the rolled alloy compared to that of the annealed one.

### 3.3 Corrosion of Mg‐based biomaterials induced production of H_2_ gas

The intrinsic ability of Mg and its alloys to degrade has resulted in a multitude of medical applications. However, in a corrosive environment, Mg undergoes anodic polarization (Equation 1), which is accompanied by the cathodic reaction of H_2_O (Equation 2), thereby generating hydrogen gas about 1 ml by the degradation of every 1 mg of Mg.^103^ In fact, the degradation of Mg‐based biomaterials followed by the production and accumulation of hydrogen gas can lead to the formation of gas cavities in the neighboring tissues, separation of corrosion products from the surface of Mg, brittle fracture of the biomaterial, and necrosis in the surrounding tissue.^104^, ^105^ In a worst‐case scenario, a considerable quantity of H_2_ gases might spread through the blood circulatory system, inducing embolism and death.^105^ However, the risk of H_2_ evolution for human health directly depends on the temporal and spatial rate of its release. In fact, it is not harmful to human health as long as the release rate would be 0.01 (ml/cm2)/day. In this case, H_2_ gases can be transported away from the place of its production, and the local buildup of a large volume of gas will not occur.^106^

From the thermodynamic point of view, in all corrosion reactions (electroneutrality requirement), when the kinetics of the anodic and cathodic reactions are equal, the rate of the accumulation of hydrogen may be affected by several measures, which decelerates the rate of either anodic reaction or the evolution reaction of H_2_. Generally, the surroundings, surface modifications, and alloying can have influences on the rate of both cathodic and anodic reactions. For example, H_2_ evolution is affected by the presence of several alloying elements or impurities^91^ with different concentrations^107^ in the alloy. The rate of H_2_ release is increased in contact with the surface of some metals known as small overpotentials such as Pt, Fe, and Ni, whereas it is decreased by several elements known as large overpotentials such as Hg and Zn.^91^ Because these elements are nobler than magnesium, intermetallic phases and impurity particles perform as local cathodes coupled with the magnesium matrix, which plays the role of the anode.^108^

**FIGURE 5:**
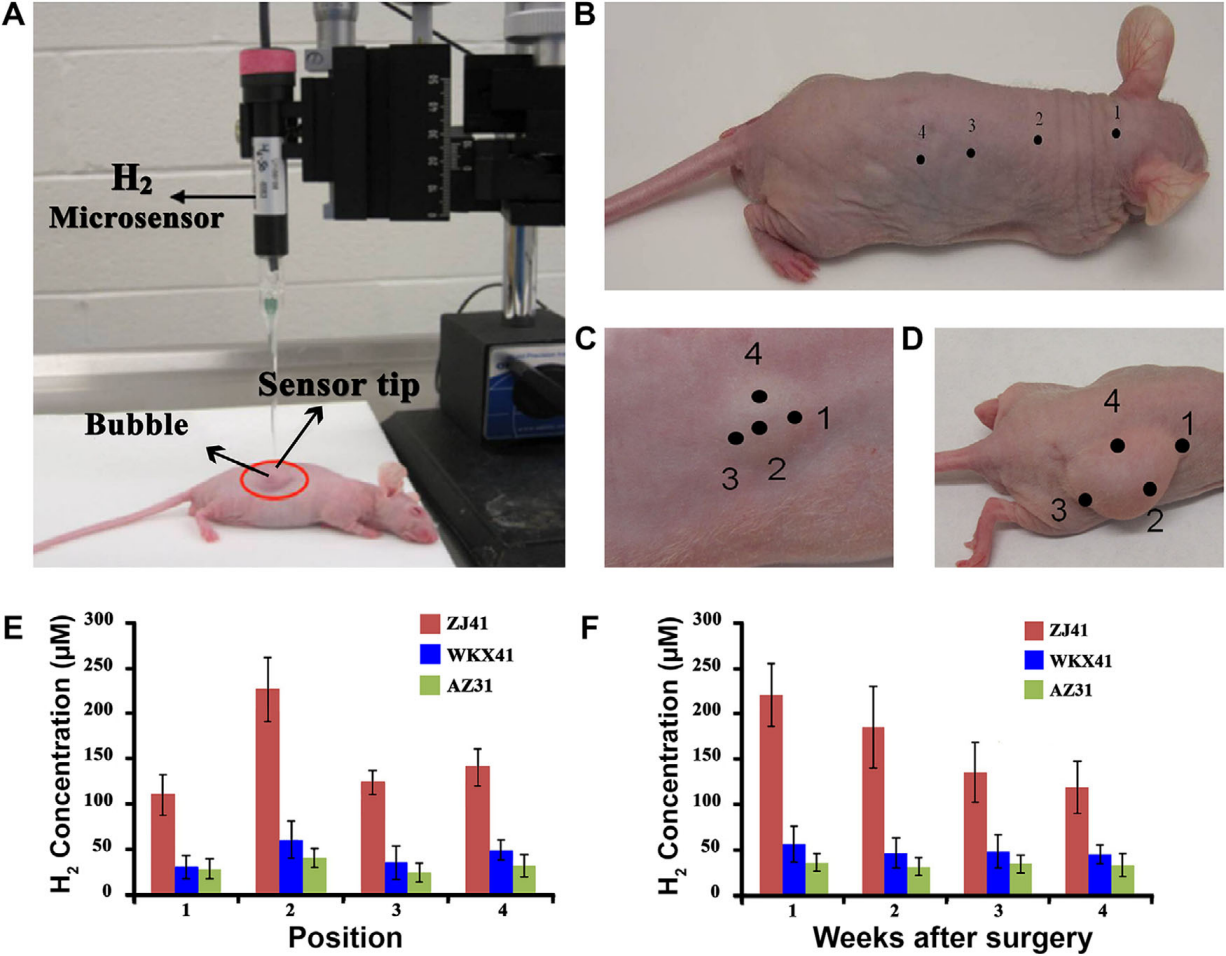
A comparative image of hydrogen evolution behavior of three different Mg alloys during corrosion. Evaluating hydrogen gases accumulated in the body of mice through implanting three Mg alloy (AZ31, WKX41, and ZJ41) discs with different corrosion resistance in mice by (A) a hydrogen microsensor. A week after surgery, the results indicated that (B) AZ31 Mg alloy with the highest corrosion resistance produced H_2_ gas too slowly to show a visible gas cavity. (A and B) Adopted with permission.^110^ (C) WKX41 Mg alloy with moderate corrosion resistance generated a smaller H_2_ cavity in comparison with (D) ZJ41 Mg alloy with high corrosion rate, which formed a big gas cavity. (E) H_2_ concentration on each point from a calibration curve demonstrated that among these three alloys, the ZJ41 alloy presented the highest corrosion rate. (F) Hydrogen concentration was evaluated weekly over a 4‐week study, which indicated that the maximum H_2_ concentration produced by these alloys and the percentage decreased in hydrogen both declined in the order of ZJ41 > WKX41 > AZ31. C‐F Adopted with permission^109^

In a study conducted by Zhao et al.,^109^ the effect of alloying on the accumulation of H_2_ gas was evaluated by the implantation of AZ31, WKX41, and ZJ41 Mg alloy discs in mice^110^ (Figure 5). As it was expected, AZ31, WKX41, and ZJ41 with slow, intermediate, and fast corrosion rates resulted in the production of gas cavities increased in the order of AZ31 (Figure 5B) < WKX41 (Figure 5C) < ZJ41 (Figure 5D) 1 week after surgery. Subsequently, the gas cavities were marked (1–4) via an H_2_ microsensor (Figure 5A) to evaluate the amount of gas accumulated in each mouse. The measurements indicated that AZ31 generated H_2_ gas very slowly, but ZJ41 alloy rapidly produced a large gas cavity in comparison with WKX41 alloy (Figure 5E). The results of this research clearly indicate the significant role of alloying elements on the corrosion behavior and H_2_ evolution of Mg‐based biomaterials; however, more studies are still needed to evaluate the role of different surface treatments and environments on the accumulation of hydrogen gas in vivo.

### 3.4 Corrosion of Mg‐based biomaterials induced pH changes

Mg‐based biomaterials have raised concern about hydroxide ions generated from the cathodic reaction of Mg (Equation 2) in a corrosive environment. This is because the production of hydroxide ions can shift the pH level into the alkaline region and might have adverse influences on local cell functions or even cell death.^111^ In reality, cathodic reactions occur in all metal corrosion reactions; however, an increase in the pH level might not always happen. The difference between the case of magnesium and other metals relates to the nature of the metal cations; especially, when it comes to the reactions with H_2_O. Because most of the transition metals such as Ti^3+^, Fe^2+^, Fe^3+^, and Cr^3+^ are acidic in nature, an acidic pH shift upon the hydrolysis of metal ions might be able to counterbalance the alkaline shift, resulted from the cathodic reaction if both reactions on the surface of the metal occur separated as in the case of uniform corrosion.^91^ In the case of localized corrosion, with a separation of the cathodic and anodic sites, the changes in local pH can be seen, and the anodic sites can indicate a very acidic electrolyte. However, as Mg^2+^ presents a neutral character, the hydrolysis reaction of Mg^2+^ might not neutralize the alkaline shift resulted from the cathodic reaction. Therefore, the concentration of various cations liberated in the alloy from the alloy composition can affect the hydrolysis reactions, as the alkaline shifts differ for different alloys. For example, because Al cations have an acidic nature, in the vicinity of corroding surfaces, the equilibrium pH value has been indicated to be higher for pure magnesium than for AZ91, which contains 9 wt.% Al.^91^

On the other side, it is noteworthy to mention that sometimes an increase in alkaline on the surface of a biomaterial might be beneficial for several applications because it assists the surface passivation of Mg by the generation of protective magnesium hydroxide films. By way of illustration, magnesium alloys may indicate an unexpectedly desirable corrosion behavior under atmospheric exposure where thin electrolyte films on the surface of the alloy enable severe alkalization. Furthermore, surface alkalization can cause self‐inhibited propagation of magnesium corrosion in any type of “occluded cells” with a confined exchange of the solution with the bulk environment. Conversely, metal cation hydrolysis reaction in most other alloys can cause acidification of the local surface, so aggressive conditions in occluded cells will arise, which can speed up the localized corrosion.^91^, ^93^

Although many other parameters may significantly or slightly change the local pH around a corroding biomaterial, the research in this field is limited. As the pH changes in the vicinity of a corroding biomaterial in vivo are hard to evaluate, experiments are often conducted under static conditions. However, the local surface chemistry evolves with time, and the hydrodynamic conditions such as blood flow around the surface of an implant can transport OH^–^ anions away from the surface.^112^ Also, in the body fluids, insignificant changes in the pH are usually observed due to buffering. Accordingly, a great deal of research is required for understanding the effect of different parameters on the pH values around a corroding biomaterial.

### 3.5 Biocompatibility of Mg and its corrosion products in the human body

Everybody knows that all biomaterials used in human beings have to be biocompatible.^113^ For this reason, the degradation products of Mg and its alloys, including H_2_ gases, released ions (Mg^2+^, OH^‒^, and alloying elements), and peeled‐off particles, need to be safe and biocompatible to the host tissues at the lowest needs. Although magnesium‐based biomaterials typically present good biocompatibility in vivo, the corrosion rate of several Mg alloys seems to be very high. This can result in the corrosion products released in greater quantity, which subsequently may deteriorate the biocompatibility of the biomaterial.^83^

In general, magnesium is an abundant mineral in the human body. The total Mg amount is announced to be ∼20 mmol/kg of fat‐free tissue. Between 50% and 60% of Mg resided within the bone, where it is believed to produce a surface constituent of hydroxyapatite mineral component.^114^ Most of the remaining Mg is contained in soft tissues and skeletal muscle. Mg is a cofactor in more than 300 enzymatic reactions required for the structural function of mitochondria, nucleic acids, and proteins. The body needs Mg to maintain the health of the immune system, bones, teeth, and muscles.^26^ Moreover, several studies have found that elevating brain Mg will enhance learning and memory functions.^115^ On the other hand, further research is needed to unambiguously realize the way magnesium release might affect the biological surroundings.

H_2_ gas is considered a favorable medicinal agent due to its therapeutic and restrictive influences on different diseases.^116^, ^117^, ^118^, ^119^, ^120^, ^121^ It is believed that the reactive oxygen species (ROS) are reduced by hydrogen gas in tissues. Furthermore, this gas has antioxidant and anti‐inflammatory influences on ischemia–reperfusion injuries, in which the ROS are generated in excess.^122^, ^123^ Apart from these properties, several studies have indicated the advantages of inhaled H_2_ on acute myocardial infarction and out‐of‐hospital cardiac arrest.^124^, ^125^ For these reasons, the presence of H_2_ might not result in a deterioration of biocompatibility, although the correlation between the biological effects of H_2_ and Mg as a source that continuously generates H_2_ gas during the corrosion is under investigation.

Despite the negative effect of hydroxide ions on biocompatibility, recent research has shown that OH^‒^ ions generated during the corrosion of Mg can lead to an antibacterial effect. Robinson et al.^126^ studied the influence of higher concentrations of Mg^2+^ and OH^–^ ions on the in vitro growth of *Escherichia coli*, *Pseudomonas aeruginosa*, and *Staphylococcus aureus*. The results indicated that increasing the concentration of OH^‒^, in contrast to Mg^2+^, can limit the growth of these bacteria. In addition to the antibacterial property, OH^‒^ ions are reported to promote bone growth.^127^ Apart from OH^‒^, released ions from alloying elements can affect the biocompatibility of Mg alloy biomaterials. Some alloying elements might cause poisoning and adverse tissue reactions, whereas some alloying elements are toxic only in excessive amounts. It is thus essential that researchers take advantage of elements found in the human body for Mg alloy biomaterials.^8^, ^128^ Magnesium degradation also produces soluble or insoluble degradation products, which are nontoxic and precipitate on the surface of the Mg biomaterial.^11^, ^83^ However, more effort should be taken to clearly demonstrate the biological effects of different degradation products on human health.

## 4 OPPORTUNITIES TO OVERCOME THE CURRENT CHALLENGES

Magnesium, the lightest metal found on earth, is considered one of the potential candidates for medical applications because it has a high strength‐to‐weight ratio, good inherent creep resistance, and mechanical properties close to those of the bone.^129^ Moreover, among biodegradable metals, Mg is known to have the best biocompatibility.^130^ However, the high corrosion rate of Mg in the physiological conditions hinders the full use of its functionality. For this reason, enhancing the corrosion resistance of Mg is the only technique to overcome the abovementioned drawbacks for biomedical applications. In this way, Mg‐based biomaterials can be designed to degrade in a tailored behavior to have a controlled time‐based performance in the human body to match the needs of a specific biomaterial in a range of applications. Moreover, the corrosion products at an improved corrosion resistance will release at a slower rate, thereby enabling the host tissue to deal with them. Because of that, worldwide researchers are developing multiple ways to achieve the best corrosion resistance of Mg‐based biomaterials, without disruption of the biocompatibility, through designing new Mg alloys and surface treatment techniques.^131^

### 4.1 Selection of suitable alloying elements

It has been well noted that high‐purity Mg might exhibit higher corrosion resistance in comparison with several Mg alloys. This is because some additional phases speed up the corrosion of the alpha‐Mg matrix through micro‐galvanic corrosion, and generally trigger substantial corrosion rates. As magnesium alloys contain alpha‐Mg matrix, additional phases might contain phases associated with the impurity elements such as iron, nickel, copper, and cobalt if their concentrations are more than the (composition dependent) impurity limits.^90^ On the other hand, the addition of several alloying elements to magnesium is typically believed to have beneficial effects on the corrosion resistance, degradation rate, mechanical integrity, hydrogen evolution, and surface alkalization of Mg‐based biomaterials.^46^, ^128^ As biocompatibility is an important factor when designing an Mg alloy for degradable biomaterials, it is thus imperative that designers use alloying elements found in the body such as calcium (1100 g in the body), zinc (2 g in the body), manganese (12 mg in the body), strontium (0.3 g in the body), lithium (2–4 ng/g in blood serum), zirconium (<250 mg in the body), yttrium and lanthanides (<47 μg in blood serum).^8^ However, the concentration of alloying elements utilized in the production of different Mg alloy biomaterials should be assessed because toxic elements (e.g., Al) may induce no deleterious effects in an adequate low concentration, whereas excess amounts of essential trace elements (e.g., Fe) can cause poisoning.^128^ As the concentration of the liberated ions at a particular position of the tissue around the biomaterial differs with tissue reconstruction processes of the host as a function of time, the local microenvironment (e.g., the local blood supply), and space (the distance to the biodegradable Mg biomaterial), it is essential that designers control the local concentration of the released ions from Mg alloys below their permissible limits for the design of the final biodegradable biomaterial made of Mg alloys.^8^ Table 2 summarizes the biological and metallurgical characterization and the toxicology of commonly used alloying elements found in the human body.

**TABLE 2:** Biological and metallurgical characterization, as well as toxicology, of alloying elements for magnesium biomaterials

Alloying elements	Biological characterization of alloying elements	Metallurgical characterization of alloying elements	Toxicology	References
Ca	Ca is a key nutrient in the human body. It helps the growth and maintenance of bones, healthy teeth, cell function, muscle contraction, regulating heartbeat, blood clotting, decreasing blood pressure, and nerve impulse. Ca deficiency causes osteoporosis and other diseases.	The addition of Ca to Mg alloys can enhance the elongation, creep resistance, hardness, and strength of the alloys. Excessive addition of Ca results in a deterioration of corrosion resistance, as a result, Ca concentration in Mg alloys should be less than 1%.	Too much Ca in the blood can create kidney stones, weaken bones, and interfere with how the heart and brain work.	^8^, ^136^, ^140^, ^141^
Zn	Zn helps in the normal functions of many enzymes; promotes wound healing; improves neurotransmission and synapse formation; supports protein, DNA synthesis, and the sense of taste and smell; and enhances immune activity. The deficiency of Zn leads to a delayed response to both T cell‐dependent and T cell‐independent antigen.	Zn at a content below 5% enhances the corrosion resistance and strength of Mg alloys; reduces the accumulation of H_2_ gases; and improves the mechanical properties. The addition of Zn up to 3% in binary Mg alloys decreases the grain size.	Zn^2+^ can form ZnCl_2_, which might damage partial cells lining of the stomach. Excessive absorption of Zn is neurotoxic and can impede bone growth.	^17^, ^142^, ^143^
Mn	Mn is a necessary nutrient for intracellular activities. It plays a positive role in blood clotting, energy production, antioxidant defense, digestion, immune response, and regulation of neuronal activities. It helps the normal functionality of the brain, nervous system, growth of cells, and cellular homeostasis.	Small additions of Mn in Mg alloys can improve corrosion resistance without changing the mechanical properties; reduce elongation and ultimate yield strength for binary Mg‐Mn alloys. In Mg alloys, Mn is usually limited to below 1% wt.	Overexposure to Mn might result in a neurological disorder.	^144^, ^145^, ^146^
Sr	Sr is a natural bone‐seeking element that accumulates in the skeleton due to its close physical and chemical properties with Ca. It can decrease bone resorption; stimulate the growth of osteoblasts; and enhance bone strength and bone mineral density. Moreover, the degradation of Mg–Sr alloys helps to deposit Sr‐substituted HA, which is beneficial for bone mineralization.	Sr is a grain size refiner. The addition of Sr into Mg alloys tends to form Mg–Sr phases that distribute along grain boundaries. The concentration of Sr below 2 wt% enhances the strength and corrosion resistance and decreases ultimate compressive strength and ultimate strain, whereas excessive Sr addition leads to weaker mechanical properties and a higher corrosion rate.	Excessive Sr might increase the risk of blood clots and skeletal abnormalities.	^147^, ^148^, ^149^, ^150^
Li	Li is one of the most extensively used elements in the treatment of manic depressive psychoses. Although defined Li deficiency diseases are still unknown, studies indicate that low Li intake from water supplies was associated with increased rates of suicides, homicides, and arrest rates for drug use and other crimes.	Adding Li into pure Mg can slightly decrease the grain size, resulting in the distribution of Mg–Li phases with bcc structure along grain boundaries. By forming bcc structural phases, deformability increases through the addition of Li (>11%). On the other hand, adding Li at a concentration of less than 9% can improve the corrosion resistance.	An excessive amount of Li might cause central nervous system disorders.	^151^, ^152^, ^153^, ^154^
Zr	Zr indicates osseointegration and great biocompatibility both in vitro and in vivo. This element mostly accumulates in the skeleton than in tissue.	The addition of Zr less than 2% in Mg alloys improves the corrosion resistance, mechanical properties, elongation, ductility, ultimate yield strength, and specific damping capacity, which might assist the absorption of vibrations and stresses at the interface between bone and implant. Moreover, this element is a great grain refiner.	Generally, Zr shows low systemic toxicity. However, it should be used with scrutiny according to the applied dosage.	^29^, ^136^, ^155^
Y and rare earth element (REEs)	Y and REEs are present in low abundance in organisms and water bodies. They are not necessary elements for organisms. However, some of them have the potential for cancer treatment.	In general, REEs in Mg alloys can enhance electrochemical behavior, corrosion resistance, mechanical properties, and creep resistance. Furthermore, they are good grain refiners.	Ce and La indicate the highest cytotoxicity. Besides, Ce and Pr cause hepatotoxicity.	^136^, ^156^, ^157^

It is reported that aluminum in Mg alloys has a beneficial effect on corrosion resistance, mechanical integrity, surface alkalization, and hydrogen evolution, although this metal within the human body develops neurotoxicity and Alzheimer's disease.^132^ As a consequence, Al is not suitable for biomedical applications, and it had better be eliminated from the design of Mg alloy biomaterials.^46^ As calcium, zinc, and manganese are vital for human health, these elements should be the first option for designing biomaterials made of Mg alloys.^133^ Hou et al.^134^ studied the biocompatibility, corrosion behavior, and mechanical properties of biodegradable Mg–3Sn–1 Zn–0.5Mn alloys. The in vivo results indicated that the implanted alloy in the femoral shaft and the dorsal muscle of the rabbit exhibited great biocompatibility. Moreover, they revealed that this as‐extruded alloy was a promising material for biodegradable implants due to their improved corrosion resistance and mechanical properties. In another study, Jiang et al.^135^ studied and compared the cytocompatibility and degradation behavior of four binary MgSr alloys (Mg–*x*Sr, *x* = 0.2, 0.5, 1, and 2 wt.%) as well as four ternary MgCaSr alloys (Mg–1Ca–*x*Sr, *x* = 0.2, 0.5, 1, and 2 wt.%) using direct culture with bone marrow‐derived mesenchymal stem cells. They indicated that Mg–1Sr and Mg–2Sr alloys had the lowest degradation rates compared to the other binary MgSr and ternary MgCaSr alloys. Ternary MgCaSr alloys indicated enhanced adhesion of bone marrow‐derived mesenchymal stem cells in comparison with binary MgSr alloys except for the Mg–1Ca–0.2Sr alloy. Finally, Mg–1Sr, Mg–1Ca–0.5Sr, and Mg–1Ca–1Sr alloys were selected as the best alloys between all the mentioned alloys due to their overall performances in terms of cytocompatibility and degradation.

Mg alloys with limited concentrations of Zr and Y are also well‐known for being suitable for biomedical applications. This is because the human body contains Zr and Y in tiny amounts. In addition to that, Zr generally has low toxicity.^136^ Chou et al.^137^ implanted WZ42 (Mg–4.0%Y–2.0%Zn–1.0%Zr–0.6%Ca in wt.%) alloy as wires rolled around the outside of the femurs as a cerclage and as pins to repair an osteotomy in rat femurs. The results indicated that the WZ42 alloy could be a potential material for load‐bearing orthopedic applications because normal bone healing occurred in femurs fixed with the WZ42 alloy after 14 weeks of surgery, and no accumulation of magnesium, as well as alloying elements, was observed in the liver and kidney of rats.

Another important issue about finding suitable alloying elements for Mg‐based biomaterials is that designers should find the most appropriate alloying elements for a specific application. For instance, for designing vascular stents, Mg–Li‐based alloys are of great interest. As these alloys possess good ductility, they can fulfill the requirements of expandable vascular stents.^29^ Wu et al.^138^ fabricated two multiphase extruded rods made of Mg–6Li–1 Zn (LZ61) and Mg–9Li–1 Zn (LZ91) alloys to implant them in mice. Measurements indicated that both LZ61 and LZ91 alloys showed great biocompatibility and more than 40% elongation at fracture. However, it is reported that LZ61 alloy exhibited a better balance of mechanical properties, biocompatibility, and corrosion resistance in comparison with LZ91 alloy, showing its potential for cardiovascular stent application.

Alloying design of biodegradable Mg as promising orthopedic biomaterials should have adequate amounts of calcium, manganese, and strontium. The reason for this is because these alloying elements can promote the formation of new bone and accelerate the bone healing process. Xia et al.^139^ evaluated the mechanical strength, corrosion behavior, and cytocompatibility of ternary Mg–(3.5 and 6.5 wt%) Li–(0.2, 0.5, and 1.0 wt%) Ca alloys. The results indicated the better mechanical strength of these alloys in comparison with high purity Mg. Then, they implanted Mg–3.5Li–0.5Ca alloys into the femurs of mice. The result of in vivo tests showed that these alloys did not cause any adverse effects and thickness of the cortical bone around the Mg–3.5Li–0.5Ca alloy rods had significantly increased, indicating Mg–3.5Li–0.5Ca alloy a promising candidate for orthopedic applications.

As it was indicated in this text, the role of alloying elements on the biofunctionality of Mg alloy biomaterials is undeniable, although a flawless alloying system with the optimized property profile is yet to be verified.

### 4.2 Surface modification techniques

A suitable design of a biomaterial is aimed to provide the requisite biofunctionality, durability, and biological responses. The durability and biofunctionality of biomaterials are mainly controlled by the bulk properties, whereas biological responses depend on the surface roughness, topography, energy, wettability, and chemistry.^158^ It is well‐known that the surface of biomaterials can play an important role in biological interactions and tissue biocompatibility. For instance, the surface morphology has outstanding and direct influences on the cell functions and behavior, whereas cell adhesion on the wetted superhydrophobic and superhydrophilic surfaces is higher than those on the other surfaces, comprising nonwetted superhydrophobic surfaces.^159^ The interactions between the biomaterial and the physiological environment occur on the surface of biomaterials. The first biological responses of tissues to a foreign biomaterial also depend on the biomaterial surface properties. Therefore, proper surface treatment on the surface of Mg biomaterials can enhance surface properties while preserving the bulk attributes^160^ and surface treatment might be the key to overcoming the challenges of Mg‐based biomaterials.

**FIGURE 6:**
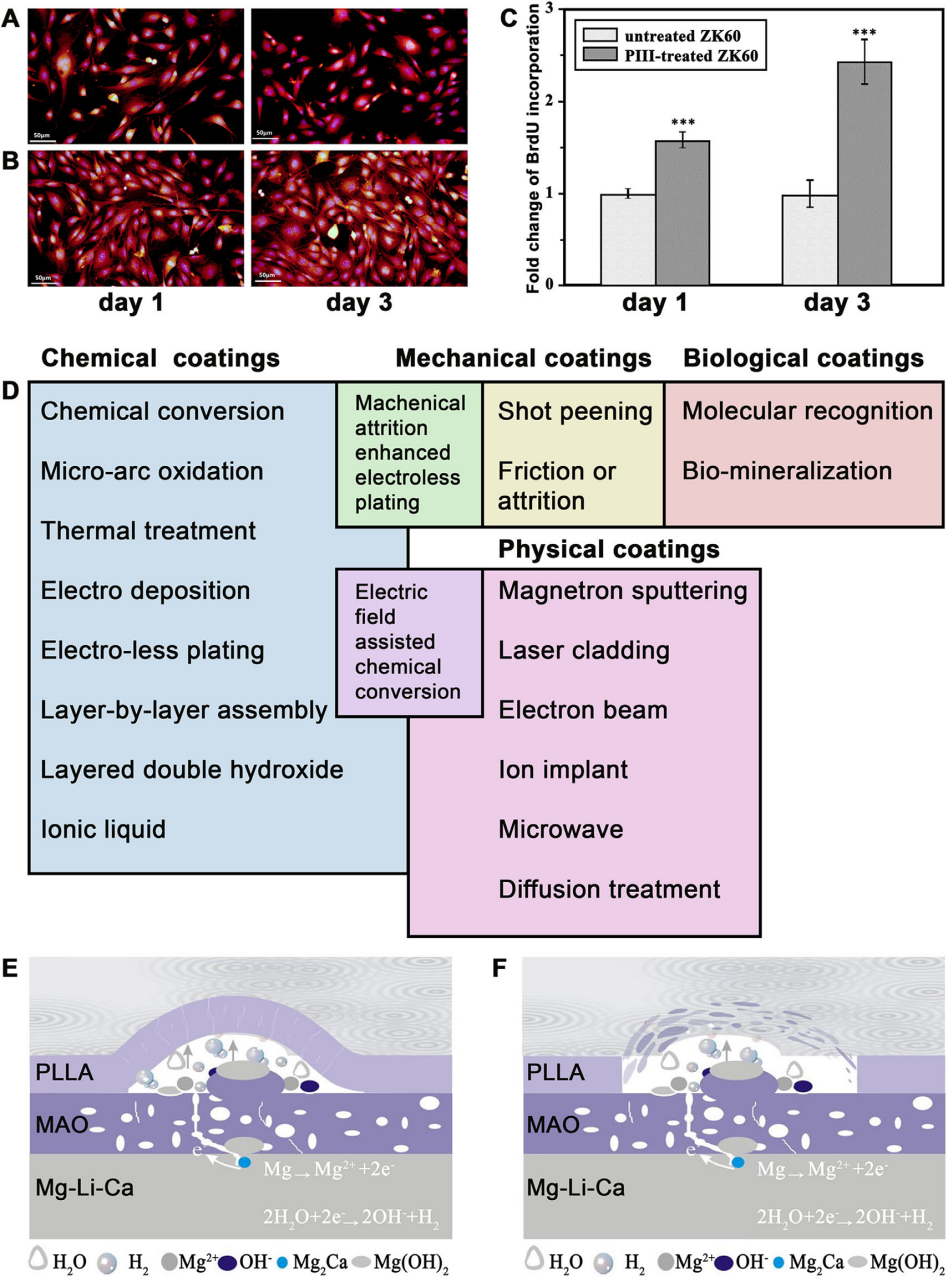
Surface modifications of Mg alloys for biomedical applications. (A) Fluorescence photographs of MC3T3‐E1 preosteoblasts cultured on the surface of untreated and (B) PIII‐treated ZK60 alloy substrates for 1 and 3 days indicating that although both untreated and treated ZK60 Mg alloys were biocompatible, the higher MC3T3‐E1 preosteoblasts adhesion occurred on the PIII‐treated ZK60. (C) The fold change of BrdU incorporation after culturing MC3T3‐E1 preosteoblasts for 1 and 3 days with untreated and PIII‐treated ZK60 substrates demonstrated that the amount of incorporation of BrdU in the PIII‐treated ZK60 was 1.7‐fold and 2.5‐fold higher at 1 and 3 days, respectively, showing better osteoblasts viability and proliferation due to the controlled release of magnesium ions from treated ZK60 alloy. Adopted with permission.^168^ (D) Schematic of coatings on the surface of Mg and its alloys. (E) Schematic demonstrations of degradation mechanism of MAO/PLLA composite coatings on the surface of Mg–1Li–1Ca alloys in Hanks' Balanced Salt Solution (HBSS) indicating the corrosion of the substrate at the initial stage and swelling of PLLA polymer on the surface of Mg alloy and (F) the final peeling‐off of the PLLA coating on the surface of the alloy under the pressure of corrosion products and hydrogen gas. Adopted with permission^176^

Generally, there are three classes of surface modification systems: the addition of a distinct layer of a material to the original surface, modification of the surface of a biomaterial by changing its microstructure or composition or both, and a mixed treatment.^161^ These systems can offer the desired route to tailor the initial reactions of the surface of Mg alloys with the physiological condition to control the corrosion, degradation, biocompatibility, and mechanical properties of Mg alloy biomaterials.^91^ Many techniques have been utilized to enhance the corrosion resistance,^162^, ^163^ bioactivity, biodegradability,^164^, ^165^ and biocompatibility^166^, ^167^ of Mg biomaterials. Lin et al.^168^ constructed multifunctional titania‐based nanolayer on the surface of as‐cast ZK60 alloy (Mg–6 wt% Zn–0.5 wt% Zr) using Ti and O dual plasma ion immersion implantation (PIII) method. Figures 6A and 6B indicate the fluorescent photographs of MC3T3‐E1 preosteoblasts adhesion on the surface of both untreated and PIII‐treated ZK60 alloys. As is presented, after 1 day of incubation no toxic effect to preosteoblasts was occurred by both untreated and PIII‐treated samples. However, on day three, an improved preosteoblast adhesion was seen on the PIII‐treated samples compared to the untreated ZK60 because of the controlled release of Mg^2+^. Moreover, according to the cell proliferation test (Figure 6C), in the PIII‐treated ZK60 group, the amount of BrdU incorporation exhibited considerably 1.7‐fold and 2.5‐fold increase, resulting in enhanced osteoblasts viability, cell attachment, and cell proliferation.

According to the coating formation mechanisms, coatings for Mg alloys can be classified into two categories: conversion and deposited coatings. Conversion or in situ grown coatings are generated by particular reactions between Mg substrate and the environment. The surface of the Mg substrate is normally converted into an oxide or passive layer through a chemical or an electrochemical procedure. This oxide layer on the surface of Mg grows inward and outward at the same time, resulting in geometry changes of the component. These formed layers, which are inorganic, indicate a ceramic‐like character. On the other hand, Mg substrates do not participate in the production of deposited or ex situ coatings, which consist mainly of organic‐based materials. Moreover, the binding force between the surface of Mg and its coating is guaranteed by intermolecular and mechanical forces. These coatings are typically regarded as the outmost layer or functional layer due to their weak adhesion with the metal substrates and they are not appropriate for the intermediate layer. Owing to the high surface alkalinity for most Mg alloys, special surface treatment is usually essential before the application of an organic coating.^33^

Coatings can be applied on the surface of Mg via chemical, physical, mechanical, and biological or biomimetic techniques (Figure 6D).^169^ In fact, based on the target application, requisite degradation rate, and cell responses, a coating can be prepared on the surface of the Mg biomaterial. For instance, for orthopedic applications, calcium phosphate–based coatings come highly recommended because these coatings can improve cytocompatibility, bioactivity, osteogenesis, and osteoconductivity of Mg‐based orthopedic implants.^170^ Another example would be some temporary biomaterials intended to degrade in a short period. In this case, it is not necessary to prepare high corrosion–resistant coatings on the surface of them.

From another point of view, for medical magnesium alloys based on functions, there are various kinds of coatings: self‐healing, self‐sacrificing, self‐cleaning, bio‐adaptable, biocompatible, biodegradable, bioactive, antibacterial, and drug‐loading coatings.^169^ It is worth noting that some coatings, on the basis of their compositions, can serve more than one function. For example, anodization is an appropriate surface treatment technique to enhance corrosion resistance and bioactivity of biomaterials. However, a single coating on the surface of Mg alloys cannot present excellent adhesion, corrosion performance, biodegradability, bioactivity, and so on. This leads to the development of multilayer coatings on the biomaterial. By way of illustration, a large number of polymer coatings have been used to protect Mg biomaterials against corrosion, yield diverse functional properties, and enhance biocompatibility, such as, polycaprolactone, poly(lactic‐co‐glycolic) acid, polylactic acid, polydopamine, collagen, and chitosan,^171^ although polymer coatings exhibit poor adhesion to the metal substrate. On the other side, micro‐arc oxidation coatings suffer from micropores and cracks, which severely limit their corrosion protection ability.^172^ Therefore, recent research suggests that designers produce a duplex coating including an inner film of micro‐arc oxidation coating followed by polymer deposition coating, as the top layer on the surface of micro‐arc oxidation coating, thereby overcoming the limitations of MAO and polymer coatings.^173^, ^174^, ^175^ Zeng et al.^176^ constructed a composite coating made of MAO/PLLA on the surface of Mg–1Li–1Ca alloy via dip‐coating followed by freeze‐drying (Figures 6E and 6F). They indicated that the micro‐arc oxidation/poly‐L‐lactic acid (MAO/PLLA) composite coating considerably reduced the corrosion rate of Mg–1Li–Ca alloy. These days many researchers from all over the world search for ways to control the biofunctionality of magnesium and its alloys and to add new functions to coatings using novel materials and techniques. However, it is still an open challenge for researchers to develop.

## 5 CONCLUDING REMARKS AND FUTURE PERSPECTIVE

The combination of biodegradability, biocompatibility, and mechanical properties similar to those of human bones renders Mg‐based alloys as promising materials for load‐bearing applications in the biomedical fields. Mg‐based implants and scaffolds not only provide mechanical support for tissue healing but also eliminate the need for implant removal surgery after the healing process. At present, Mg‐based biomaterials can be divided into three groups: Mg‐based vascular stents, Mg‐based orthopedic implants, and Mg‐based wound closure devices. Moreover, there is an increasing demand for developing new degradable biomaterials made of Mg and its alloys with new biomedical applications because studies have shown that biocompatible magnesium alloys are suitable degradable biomaterials for use in the body. On the contrary, the major drawback of magnesium is its rapid corrosion, which limits its clinical application. This rapid corrosion subsequently can lead to unexpected degradation, structural failure, hydrogen evolution, and an alkaline pH shift in the vicinity of the corroding sites. In addition, the release of corrosion products in high concentrations can affect the biocompatibility of these biomaterials. Accordingly, controlling the corrosion behavior of Mg‐based biomaterials in vivo is a significant challenge.

Extensive studies on this subject have revealed that the corrosion behavior of Mg biomaterials can be enhanced by alloying and surface treatment. The first technique is to design these biomaterials with suitable alloying elements, which are nontoxic and improve the metallurgical properties of the Mg biomaterial. The second technique is to modify the surface of Mg alloy biomaterials. Although these techniques can significantly overcome the challenges faced by magnesium biomaterials, finding the best method that ideally controls their corrosion behavior remains an open challenge for researchers to explore. As a matter of fact, the properties of Mg biomaterials hardly ever satisfy all requirements of a specific application. Apart from this, in the progress of new magnesium alloy biomaterials, testing and assessment are extremely necessary to demonstrate the efficacy of the modified biomaterials and there is often a big gap between the results obtained from in vitro and in vivo tests due to the differences in the environmental conditions. In fact, animal and human studies provide more accurate information about the behavior of Mg implants in the physiological environment. However, in vivo studies are not always performed to evaluate the corrosion rate of modified Mg alloys. Moreover, in vivo studies from the complete degradation of modified Mg alloys are rare. As a result, a great deal of investigations is still required for the development of novel biodegradable biomaterials made of Mg and its alloys.

## AUTHOR CONTRIBUTIONS

SA wrote the first draft of the manuscript. MM corrected and edited the final version of the manuscript.

## ETHICS STATEMENT

Not applicable.

## CONFLICT OF INTERESTS

The authors declare no conflict of interest.

## Data Availability

All the presented information in this article is accessible by contacting the corresponding author.
